# Seropositivity of selected chronic infections and different measures of obesity

**DOI:** 10.1371/journal.pone.0231974

**Published:** 2020-04-22

**Authors:** Dennis Freuer, Jakob Linseisen, Tim Waterboer, Frank Pessler, Carlos Alberto Guzmán, Nina Wawro, Annette Peters, Christa Meisinger

**Affiliations:** 1 Chair of Epidemiology at UNIKA-T Augsburg, Ludwig-Maximilians Universität München, Augsburg, Germany; 2 Independent Research Group Clinical Epidemiology, Helmholtz Zentrum München, German Research Centre for Environmental Health, Neuherberg, Germany; 3 Infections and Cancer Epidemiology, German Cancer Research Centre (DKFZ), Heidelberg, Germany; 4 Research Group Biomarkers for Infectious Diseases, TWINCORE, Centre for Experimental and Clinical Infection Research, Hannover, Germany; 5 Department of Vaccinology and Applied Microbiology, Helmholtz Centre for Infection Research, Braunschweig, Germany; 6 Centre for Individualised Infection Medicine, Hannover, Germany; 7 Institute of Epidemiology, Helmholtz Zentrum München, German Research Centre for Environmental Health, Neuherberg, Germany; San Raffaele Roma Open University, ITALY

## Abstract

The impact of sex-specific body fat distribution on the susceptibility to five chronic infections, helicobacter pylori and human herpesviruses 3 to 6 (i.e. varicella-zoster, Epstein-Barr, cytomegalo- and human herpesvirus 6), has not previously been examined. In the present study, seropositivity was determined via multiplex serology in serum samples of study participants collected in 2006/08 and 2013/14 during the follow-up examinations F4 (n = 3080) and FF4 (n = 2279) of the German population-based baseline KORA S4 survey. We quantified the severity of overall and abdominal obesity by body mass index, body adiposity index, waist circumference, waist-to-hip ratio, and waist-to-height ratio. Using sex-specific logistic spline-models, cross-sectional and longitudinal associations between obesity measures and seropositivity of the previously mentioned infections were investigated. Overall and abdominal fat content were significantly associated with seropositivity of varicella-zoster virus in both cross-sectional and longitudinal analyses among women. In addition, a non-significant inverse relationship with Epstein-Barr virus seroprevalence in both sexes and a trend towards a positive association with human herpesvirus 6 seropositivity in women were observed. Therefore, in women total body fat may be associated with VZV-seropositivity and may influence the reactivation of the varicella-zoster virus, independent of adipose tissue distribution.

## Introduction

There is emerging evidence that obesity, a known risk factor for several chronic diseases, may also be associated with infectious diseases [1, 2]. Although the underlying mechanisms are not entirely clear, obesity-related immune system dysregulation has been proposed to increase susceptibility to infections [3]. Prior studies investigating the association between obesity and infection risk in adults showed controversial results [4–6]. This might be partialy due to the fact that in prior studies, mainly body mass index (BMI) was used to define obesity. However, BMI as a measure of excess body weight has several limitations[7]. Furthermore, categorisation of BMI leads to the loss of within-group information, increases residual confounding, and different cut-offs (e.g. at BMI = 25 or BMI = 30) contribute to inconsistent results. It is possible that adipose tissue distribution might influence infection risk due to immunomodulatory effects. In particular, visceral adipose tissue is highly metabolically active and as such is linked to inflammation and immunity [8].

So far, no study has examined whether body fat distribution influences seropositivity (a marker of cumulative exposure, i.e. the presence of antibodies due to past or present infection, primary infection or reactivation of latent infection) against a number of human pathogens. Thus, in the present study, we aimed to determine whether general obesity and/or specific body fat distribution contribute to the seropositivity of five selected chronic infectious diseases, namely helicobacter pylori (HP), varicella-zoster virus (VZV), Epstein-Barr virus (EBV), cytomegalovirus (CMV), and human herpesvirus (HHV) 6 in middle-aged adults from the general population. These bacterium and viruses are among the most widespread pathogens worldwide. HP is linked to gastritis and gastric ulcers, VZV to chickenpox and shingles, EBV to mononucleosis, CMV to cytomegaly and pneumonia and HHV 6 to exanthema subitum. Moreover, in connection with immunodeficiency, most of these pathogens have been linked to cancer [9, 10]. In the present study, body fat and its distribution was described by using body mass index (BMI), body adiposity index (BAI), waist circumference (WC), waist-to-hip ratio (WHR) and waist-to-height ratio (WHeiR). This comprehensive work consists of two parts. Firstly, we analysed and compared cross-sectional associations between the previously mentioned anthropometric measures and the seroprevalence of chronic infections detected by multiplex serology. Secondly, using a longitudinal approach, we investigated the impact of general and visceral obesity on a new infection or reactivation of a latent infection during a period of approximately seven-year. In contrast to previous investigations, we used all obesity indices as continuous variables in the analyses and investigated sex-specific relationships.

## Methods

### Study population

**Fig 1:**
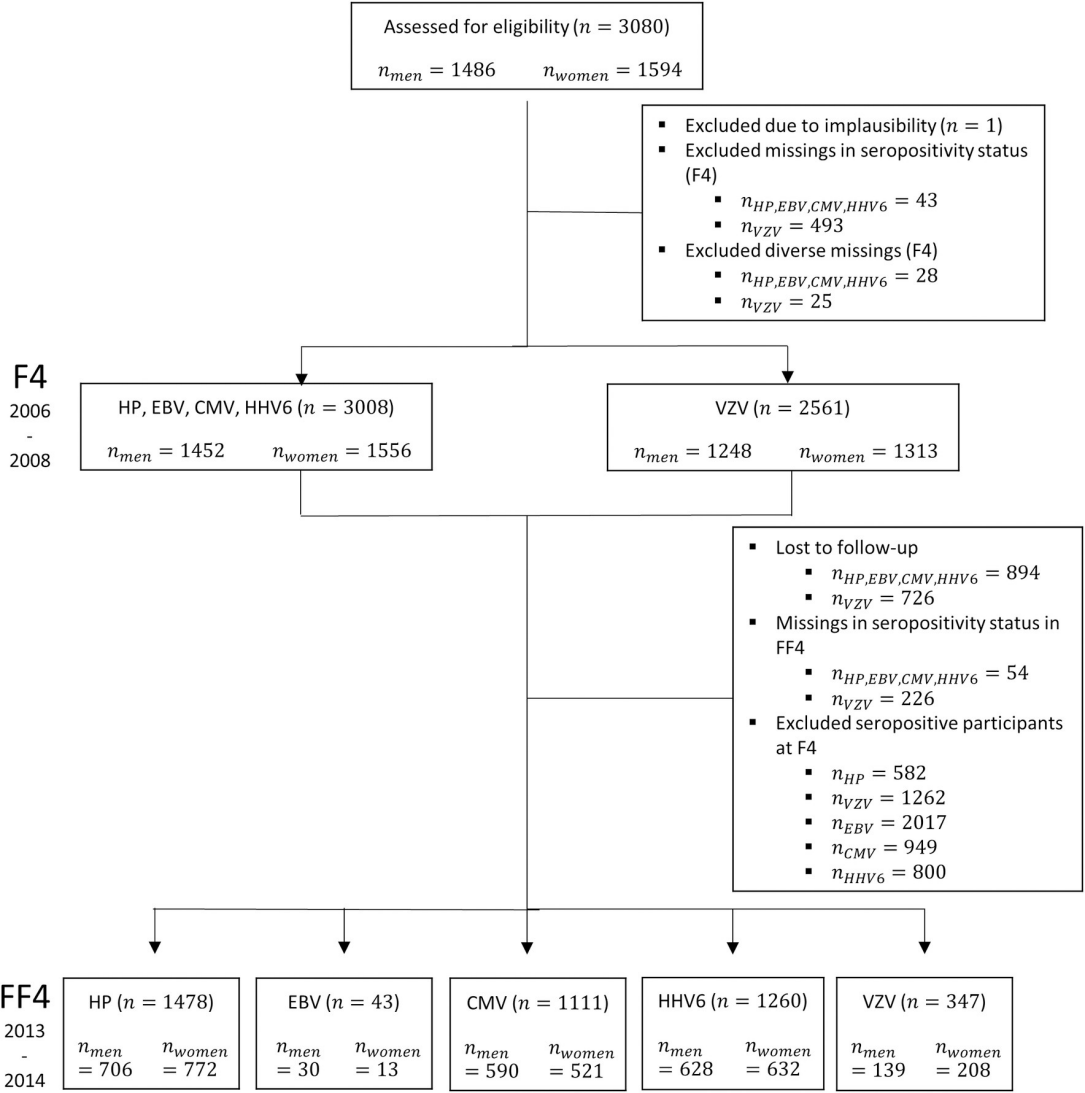
Participants of the population-based KORA cohort study included in the cross-sectional (F4) and longitudinal analyses (F4/FF4). Number of participants based on analyses for helicobacter pylori (HP), Epstein-Barr virus (EBV), cytomegalovirus (CMV), human herpesvirus 6 (HHV 6) and varicella-zoster virus (VZV).

The study included participants of the German population-based KORA (Cooperative Health Research in the Region of Augsburg) S4/F4/FF4 cohort study. The baseline study S4 was conducted in 1999/2001 (n = 4261, age 25–74 years), the first follow-up examination F4 (n = 3080) and the second follow-up examination FF4 (n = 2279) were conducted from 2006 to 2008 and from 2013 to 2014, respectively. In F4, 1,181 S4-participants and in FF4, 801 F4-participants did not participate due to death or because they moved out of the study area, refused participation, were too ill, not interested, too busy to participate, or could not be contacted. Further information on study design, recruitment and data collection procedures have been described in detail elsewhere [11]. Fig 1 specifies the study samples used in the present analyses. All participants gave written informed consent and the Ethics Committee of the Bavarian Medical Association approved the study. The investigations were carried out in accordance with the Declaration of Helsinki.

### Cross-sectional and longitudinal analyses

The multiplex measurement was conducted in all 3,080 F4-participants and all 2,279 FF4-participants. One out of 3,080 participants was excluded due to implausible measurements. Data was not available for VZV serology in 493 F4 subjects, and for the other 4 chronic infections in 43 participants; this is due to either failure of antibody analyses or different technical reasons, like pipetting error or insufficient bead count. Consequently, two different samples were used for the cross-sectional investigations (Fig 1). For the analyses of the association between anthropometric measures and the seroprevalence of VZV, 2,561 participants were included; regarding HP, EBV, CMV as well as HHV 6, the data of 3008 subjects could be used (after exclusion of further 25 and 28 observations with missing values in the remaining variables).

Regarding the longitudinal analyses, we included subjects that participated in both, F4 and FF4, and were seronegative at F4. Altogether, 1,478 participants seronegative for HP, 1,260 for HHV 6, 1,111 for CMV, 347 for VZV and 43 for EBV formed the basis for the longitudinal approach.

### Anthropometric measures (exposures) and lifestyle variables

At the study centre visits, all participants were weighed in light clothes and shoes by trained and certified examiners according to a standard operating procedure. Body height and weight were measured digitally (SECA 221, SECA 709), allowing for measurements accurate to 0.1 cm or 0.1 kg. Body fat mass and body fat distribution were described by calculating body mass index (BMI = weight in kg / height in m^2^), body adiposity index (BAI = (hip circumference in cm / height in m^3/2^) - 18) [12], waist circumference (WC in cm), waist-to-hip ratio (WHR) and waist-to-height ratio (WHeiR).

Trained medical interviewers collected information on medical history, physical activity, smoking behaviour, and alcohol consumption. Diabetes was assessed based on validated self-reported diagnosis of type 2 diabetes. Hypertension was defined using the mean of the second and third blood pressure readings with SBP ≥ 140 mmHg and/or DBP ≥ 90 mmHg, or intake of anti-hypertensive medication in participants with a physician’s diagnosis of hypertension. All detailed procedures of examination are described elsewhere in detail [13].

### Seropositivity of chronic infections (outcomes)

We focused in our analyses on participants with a successful classification of seropositivity for the five following chronic infections: helicobacter pylori and the human herpesviruses 3 to 6, also known as varicella-zoster virus, Epstein-Barr virus, cytomegalovirus and human herpesvirus 6. Antibody responses to various antigens of these pathogens were measured via multiplex serology as described previously [14]. Briefly, viral and bacterial antigens (Table 1) were recombinantly expressed as glutathione-S-transferase (GST)-tag fusion protein in E. coli and affinity-purified on fluorescently labelled glutathione-coupled beads (Luminex Corp, Austin, TX, USA). Distinctly labelled bead types, each carrying a different antigen, were mixed and incubated with serum (diluted 1:1000). Serum antibodies bound to the antigens were detected using a biotin-labelled secondary antibody (IgA/IgM/IgG) and subsequent incubation with the reporter fluorescent streptavidin-R-phycoerythrin. A Luminex 200 analyzer (Luminex Corp., Austin, TX, USA) then distinguished between the bead type and consequently the bound antigen as well as quantified the amount of bound serum antibody as the median fluorescence intensity (MFI) of at least 100 beads per type per serum. All assays were previously described and validated, and MFI values were dichotomized into seropositive and seronegative using previously defined standard cut-offs [15–17]. According to these standard definitions, infection status was defined as positive when exceeding a specific minimum number of antigens. In case of HP, at least four positive antigen reactions were needed for the classification of seropositive. For VZV, one antigen reaction was sufficient for classification while in case of EBV, CMV and HHV 6 at least 2 positive antibody responses determined seropositivity (Table 1).

**Table 1:** Specific sets of antigens and cut-offs used for identifying seropositivity of the respective infection.

Pathogen	Seropositivity	Set of antigens (seropositivity cut-offs)
Helicobacter pylori	*≥ 4* seropositive antigens	Cad (100), CagA (1800), CagD (150), CagM (120), Catalase (250), GroEl (100), HcpC (100), HP 0305 (100), HpaA (100), HyuA (150), NapA (100), Omp (250), UreA 150), VacA (180)
Varicella-zoster virus	*≥ 1* seropositive antigen	gI(ORF67) (100), gE(ORF68) (80)
Epstein-Barr virus	*≥ 2* seropositive antigens	EA-D (150), EBNA-1 (peptid) (150), VCA p18 (200), Zebra (150)
Cytomegalovirus	*≥ 2* seropositive antigens	pp28 (150), pp52 (150), pp65 (150), pp150 Nter (150)
Human herpesvirus 6	*≥ 2* seropositive antigens	IE1A trunc (50), IE1B trunc (50), p100 trunc (50), p101 K trunc (50)

### Statistical analysis

Depending on sample sizes and variance homogeneity, all P-values for numerical variables were calculated by either the two-sided t-test or Welch’s t-test, whereas the Fisher’s exact test was used for categorical data. Odds ratios regarding the associations between different anthropometric measures and seropositivity of the given infections were calculated separately using multivariable logistic models in both the cross-sectional and longitudinal approaches. All known confounders were selected using directed acyclic graphs (DAGs) in conjunction with the disjunctive cause criterion [18]. Using these criterion, variables that cause either the exposure or outcome or both were included, while mediators as well as instrumental confounding factors were excluded. Furthermore, we investigated whether the anthropometric measures were modified by sex and age, applying formal tests for interaction. If appropriate, stratified models were performed using a 5% significance threshold.

To capture the log-linearity assumption, get the best possible fit and avoid loss of information in terms of residual confounding, we used, if necessary, restricted cubic splines within the regression models with a model-specific number of knots. The optimum number of quantile-based knots (in the range of 3 to 7) was determined separately for each numerical covariate within each model by Akaike’s information criterion (AIC) using a 5% significance level as evidence for non-linearity. The shapes and odds ratios of non-linear associations between the different anthropometric measures and seropositivity of infections were presented with the lowest estimates as reference points.

Because of an extreme outlier (and therefore a wide range of values without observations) on the one hand and the zero inflation on the other hand regarding the distribution of *alcohol consumption*, a square root transformation was done with respect to the AIC. This transformation allowed a sensible placement of the spline nods and avoided mathematical problems with the non-drinkers. To handle the zero inflation of the distribution, where many participants did not drink alcohol, we separated the group of drinkers from the group of non-drinkers by including an additional dichotomous variable *drinking status* in the models. As a result, the sex-specific regression models were adjusted for *age*, *years of education* and rooted *alcohol consumption* (g/day) as numerical as well as self-reported *smoking* (smoker, ex- vs. non-smoker), *physical activity* (active vs. inactive) and *drinking status* (non- vs. drinker) as categorical variables.

All anthropometric variables were *σ*-standardized to allow for the direct comparison of the effect estimates. To judge the effect of unmeasured confounding, a sensitivity analysis was performed by determining the appropriate E-values of point estimates for significant linear associations. An E-value is a measure of the minimum significant association (e.g. odds ratio) of an unobserved confounder that is needed to explain away a significant exposure-outcome association. Finally, a missing value analysis revealed that the MCAR assumption held for missings in the seropositivity status of chronic infections, so that imputation algorithms could not be applied to the data and a complete case analysis had to be performed.

The software package R, version 3.4.3, was used for the statistical analyses. All P-values presented were for two-sided tests.

## Results

### Baseline characteristics

At baseline participants were on average 56 years old (SD: 13), 169 cm tall (SD: 10) and weighed 78.6 kg (SD: 15.4) (Table 2). Moreover, they had a mean BMI of 27.6 kg/m^2^ (SD: 4.8), BAI of 30.8 (SD: 5.9), waist-to-hip ratio of 0.9 (SD: 0.1), waist-to-height ratio of 0.6 (SD: 0.1) and reported 11.8 years of education (SD: 2.6). Fifty-two percent of the subjects were women; 30% drank no alcohol while the median-consumption of those who drank amounted to 14.5 g/d (IQR: 5.7, 26.6). Furthermore, 38% of the participants were former and 18% current smokers. Forty-six percent reported to be physically inactive. Referring to the WHO-Europe cut-offs [19], 42% of subjects were overweight (25 kg/m^2^ ≤ BMI < 30 kg/m^2^) and 26% obese (BMI ≥ 30 kg/m^2^).

**Table 2:** Baseline characteristics^a^ of participants of the population-based KORA F4 study, stratified by seropositivity of helicobacter pylori and the human herpesviruses 3 to 6, n = 3,080, 2006–2008.

	Helicobacter pylori			Varicella-zoster virus			Epstein-Barr virus			Cytomegalovirus			Human herpesvirus 6		
	seronegative	seropositive		seronegative	seropositive		seronegative	seropositive		seronegative	seropositive		seronegative	seropositive	
	n = 2,071	n = 937	P-value	n = 550	n = 2,011	P-value	n = 61	n = 2,947	P-value	n = 1,544	n = 1,464	P-value	n = 1,824	n = 1,184	P-value
Body mass index (kg/m^2^)	27.33 (4.77)	28.18 (4.72)	<0.001	27.17 (4.75)	27.77 (4.8)	0.01	28.11 (4.94)	27.58 (4.77)	0.40	27.27 (4.69)	27.93 (4.84)	<0.001	27.55 (4.63)	27.67 (5)	0.49^a^
Body adiposity index^c^	30.46 (5.83)	31.57 (5.99)	<0.001	30.97 (5.88)	30.76 (5.97)	0.48	29.84 (5.78)	30.83 (5.91)	0.20	30 (5.67)	31.65 (6.04)	<0.001^a^	30.74 (5.79)	30.91 (6.08)	0.42
Waist circumference (cm)	92.66 (13.82)	96.02 (13.17)	<0.001	91.74 (13.79)	94.51 (13.75)	<0.001	96.12 (13.61)	93.66 (13.71)	0.17	93.12 (13.73)	94.33 (13.66)	0.02	93.7 (13.48)	93.71 (14.06)	0.98
Waist-to-hip ratio	0.87 (0.09)	0.9 (0.09)	<0.001	0.87 (0.09)	0.89 (0.09)	<0.001	0.89 (0.08)	0.88 (0.09)	0.25	0.88 (0.09)	0.88 (0.09)	0.08	0.88 (0.09)	0.88 (0.09)	0.28
Waist-to-height ratio	0.55 (0.08)	0.57 (0.08)	<0.001	0.55 (0.08)	0.56 (0.08)	<0.001	0.56 (0.08)	0.56 (0.08)	0.75	0.55 (0.08)	0.57 (0.08)	<0.001	0.56 (0.08)	0.56 (0.08)	0.96^a^
Age (years)	54.06 (13.1)	60.8 (12.32)	<0.001^a^	56.68 (13.03)	56.34 (13.12)	0.59	50.67 (11.97)	56.27 (13.24)	<0.001	53.74 (13.39)	58.71 (12.58)	<0.001^a^	56.12 (13.11)	56.22 (13.43)	0.83
Alcohol consumption (g/day)	14.38 (19.28)	14.01 (19.49)	0.62	14.21 (19.11)	14.4 (19.4)	0.84	13.43 (18.75)	14.28 (19.36)	0.73	14.87 (20.02)	13.63 (18.58)	0.08	13.78 (19.01)	15.02 (19.82)	0.09
Years of education	11.96 (2.65)	11.28 (2.58)	<0.001^a^	11.83 (2.72)	11.74 (2.65)	0.45	12.38 (2.93)	11.74 (2.64)	0.06	11.97 (2.65)	11.52 (2.62)	<0.001	11.77 (2.63)	11.72 (2.67)	0.66
Physical inactive	905 (0.44)	466 (0.5)	<0.001	233 (0.42)	929 (0.46)	0.11	31 (0.51)	1340 (0.45)	0.44	658 (0.43)	713 (0.49)	<0.001	819 (0.45)	552 (0.47)	0.37
Women	1102 (0.53)	454 (0.48)	0.02	331 (0.6)	982 (0.49)	<0.001	19 (0.31)	1537 (0.52)	<0.001	719 (0.47)	837 (0.57)	<0.001	908 (0.5)	648 (0.55)	0.01
Smoking status			0.82			0.13			0.07			0.22			0.35
former	778 (0.38)	363 (0.39)		207 (0.38)	791 (0.39)		18 (0.3)	1123 (0.38)		600 (0.39)	541 (0.37)		696 (0.38)	445 (0.38)	
current	373 (0.18)	164 (0.18)		84 (0.15)	362 (0.18)		7 (0.11)	530 (0.18)		258 (0.17)	279 (0.19)		311 (0.17)	226 (0.19)	
Non-drinker	581 (0.28)	322 (0.34)	<0.001	150 (0.27)	617 (0.31)	0.13	12 (0.2)	891 (0.3)	0.09	426 (0.28)	477 (0.33)	<0.001	566 (0.31)	337 (0.28)	0.14

^a^ Data presented in form of either mean (standard deviation) or absolute (relative) frequencies. Given P-values calculated with two-sided t-tests for numerical and with Fisher’s exact tests for categorical variables.

^b^ Given P-value calculated using Welch’s t-test

^c^ Body adiposity index = (hip circumference in cm / height in m^3/2^) - 18.

All anthropometric measures were significantly higher in HP seropositive participants than in seronegative subjects (Table 2). Similar results were found in case of VZV and CMV infections except for the measures BAI and waist-to-hip ratio, respectively. However, there was no significant difference in anthropometric measures between the seropositive and seronegative groups in EBV as well as HHV 6. Furthermore, notable differences were observed regarding baseline characteristics between seropositivity and seronegativity of both the HP and CMV. Seropositive participants were significantly older, shorter, less educated, less physically active, more frequently had hypertension as well as diabetes and were more likely to drink alcohol.

**Fig 2:**
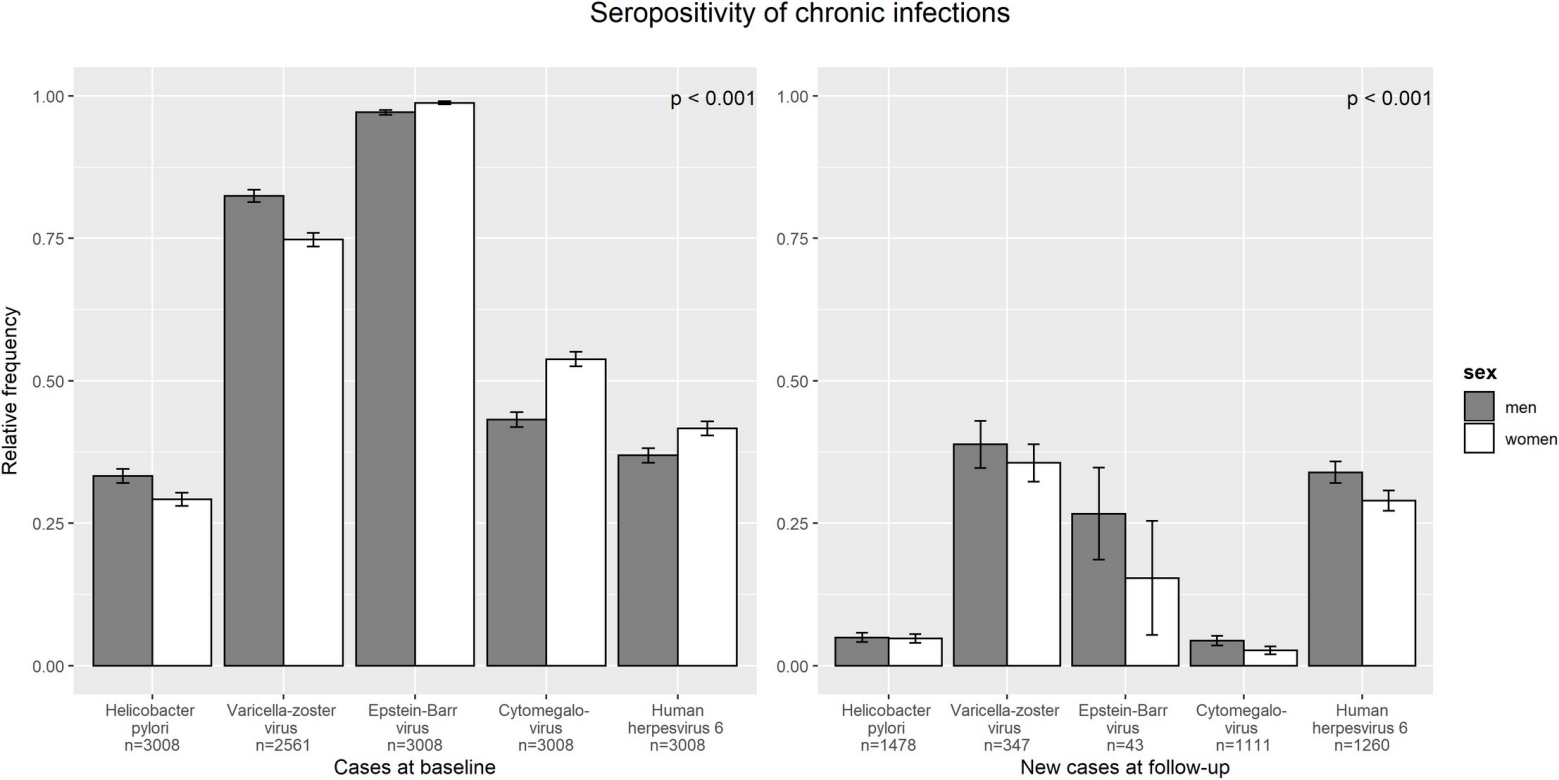
Sex-specific cross-sectional as well as longitudinal seropositivity of helicobacter pylori and the human herpesviruses 3 to 6. Error bars represent standard deviations. Note: Sample sizes differ between chronic infections. The number of infections depends on sex (*P* < 0.001).

Ninety-eight percent of the included participants with a successful multiplex antibody measurement at baseline were seropositive for EBV, 79% for VZV, 49% for CMV, 39% for HHV 6 and 31% for HP. Fig 2 reveals that VZV and HP-seroprevalence were higher in men than in women, though women showed a higher prevalence otherwise. Only three subjects (all men) were seronegative for all five of the infections.

Considering only seronegative participants at F4, 37% were observed as VZV seropositive after approximately seven years at FF4, while the proportion of seropositivity amounted to 32% in HHV 6, 23% in EBV, 5% in HP and 4% in CMV. Overall, men showed an equal or higher rate of seropositivity after 7 years than women (Fig 2). Due to the small sample size and therefore insufficient statistical power, the EBV was not included in the longitudinal investigation.

### Cross-sectional associations

**Fig 3:**
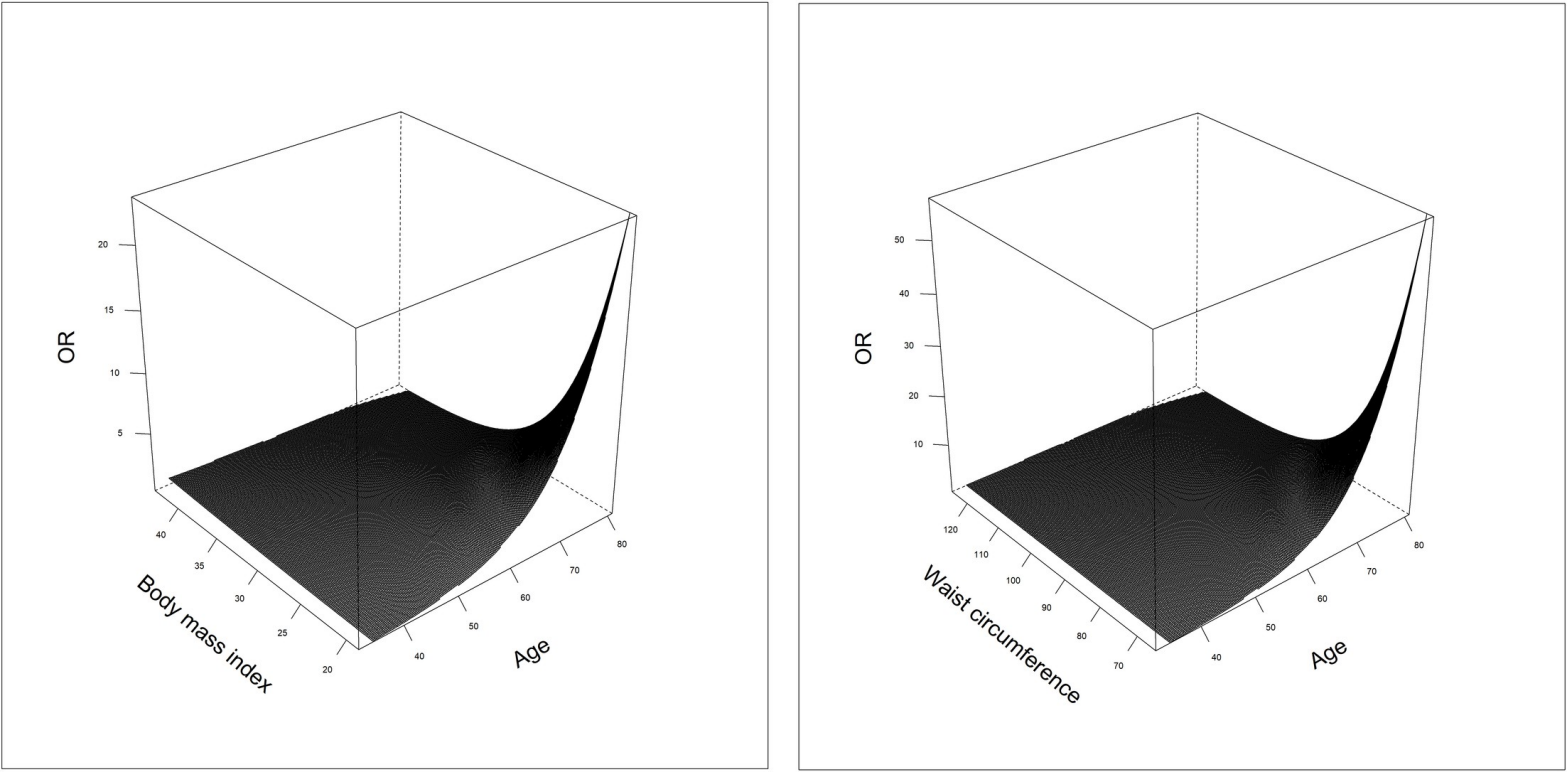
Non-linear associations with the seroprevalence of Epstein-Barr virus in women. Multivariable binary logistic models were adjusted for age, years of education, rooted alcohol consumption, physical activity, smoking and drinking status. Age modified both BMI and waist circumference in women. For reasons of clarity the 95% confidence areas were not drawn.

Since sex modified the anthropometric measures in some models, all cross-sectional as well as longitudinal analyses were stratified by sex. In men, the multivariable logistic regressions revealed consistently linear positive but non-significant associations between all investigated *σ*-standardised anthropometric measures and the prevalence of seropositivity of HP, VZV, CMV as well as HHV 6 (except BAI in HHV 6) (Table 3). The same was true for women with similar estimates regarding HP and CMV. However, all linear associations tended to be inverse for EBV-seropositivity in both sexes. Additionally, in women the associations between BMI as well as waist circumference and EBV were modified by age (Fig 3). Briefly, an increase in BMI led almost linearly to an increase of the odds ratio in middle-aged participants. The older the participants, the more the association changed to a reverse J-shaped association, where subjects with a low BMI showed a strongly increased odds ratio. Waist circumference was associated with EBV seropositivity in a similar way.

**Table 3:** Sex-specific associations^a^ of *σ*-standardised anthropometric measures with the prevalence of helicobacter pylori and the human herpesviruses 3 to 6, KORA F4 study, 2006–2008.

Men	Helicobacter pylori n = 1,452		Varicella-zoster virus n = 1,248			Epstein-Barr virus n = 1,452		Cytomegalovirus n = 1,452		Human herpesvirus 6 n = 1,452	
	OR (95% CI)	P-value	OR (95% CI)	P-value	E-value	OR (95% CI)	P-value	OR (95% CI)	P-value	OR (95% CI)	P-value
Body mass index (kg/m^2^)	1.01 (0.90, 1.14)	0.881	1.06 (0.90, 1.24)	0.505		0.81 (0.60, 1.09)	0.164	1.02 (0.92, 1.15)	0.678	1.05 (0.94, 1.18)	0.392
Waist-to-hip ratio	1.09 (0.95, 1.24)	0.218	1.08 (0.90, 1.28)	0.404		0.92 (0.64, 1.32)	0.650	1.08 (0.95, 1.23)	0.214	1.04 (0.91, 1.18)	0.595
Waist-to-height ratio	1.07 (0.94, 1.22)	0.307	1.05 (0.88, 1.25)	0.579		0.84 (0.59, 1.19)	0.316	1.08 (0.96, 1.23)	0.198	1.02 (0.90, 1.15)	0.811
Waist circumference (cm)	1.01 (0.90, 1.15)	0.841	1.03 (0.88, 1.21)	0.704		0.89 (0.64, 1.23)	0.469	1.02 (0.91, 1.15)	0.715	1.06 (0.94, 1.19)	0.347
Body adiposity index	1.07 (0.94, 1.21)	0.306	1.03 (0.87, 1.21)	0.769		0.80 (0.57, 1.11)	0.185	1.09 (0.97, 1.23)	0.158	0.97 (0.86, 1.09)	0.573
**Women**	**n = 1,556**		**n = 1,313**			**n = 1,556**		**n = 1,556**		**n = 1,556**	
Body mass index (kg/m^2^)	1.04 (0.92, 1.17)	0.525	**1.17 (1.02, 1.35)**	**0.027**	1.62	**age-interaction**^a^	**0.043**	1.07 (0.96, 1.20)	0.222	non-linear^b^	0.063
Waist-to-hip ratio	1.06 (0.94, 1.21)	0.333	1.12 (0.97, 1.29)	0.115		0.74 (0.44, 1.23)	0.246	1.08 (0.96, 1.21)	0.194	1.02 (0.91, 1.15)	0.689
Waist-to-height ratio	1.06 (0.93, 1.21)	0.366	**1.22 (1.05, 1.42)**	**0.010**	1.73	0.65 (0.39, 1.07)	0.089	1.09 (0.96, 1.23)	0.185	non-linear^b^	0.132
Waist circumference (cm)	1.06 (0.93, 1.20)	0.380	**1.22 (1.06, 1.41)**	**0.007**	1.73	**age-interaction**^a^	**0.014**	1.09 (0.97, 1.22)	0.137	1.06 (0.95, 1.18)	0.319
Body adiposity index	1.04 (0.92, 1.19)	0.513	**1.17 (1.01, 1.36)**	**0.032**	1.63	0.73 (0.45, 1.19)	0.210	1.05 (0.93, 1.18)	0.459	non-linear^b^	0.069

Abbrevations: CI, confidence interval; OR, odds ratio.

^a^ Multivariable binary logistic regression models were adjusted for age, years of education, rooted alcohol consumption, physical activity, smoking and drinking status. E-values of point estimates are presented for significant linear associations (bold).

^b^ Body adiposity index = (hip circumference in cm / height in m^3/2^) - 18.

^c^ Exposure-age-interactions are illustrated in Fig 3.

**Fig 4:**
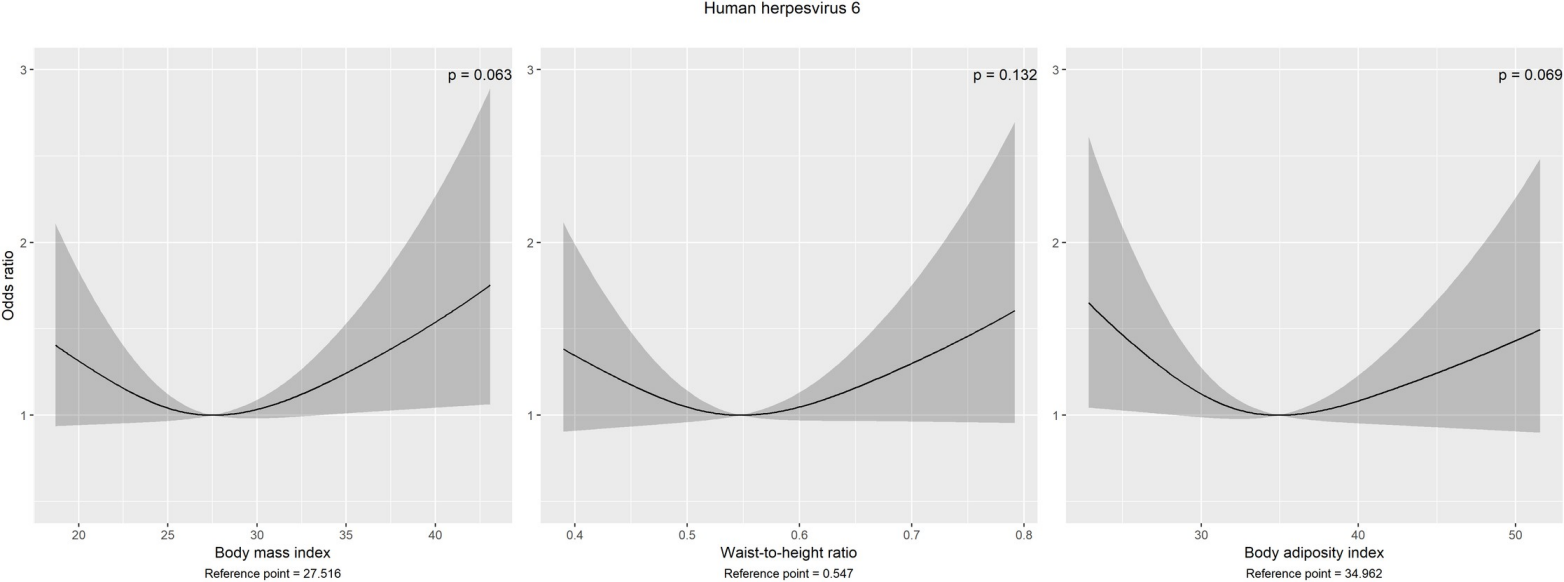
Non-linear associations with seroprevalence of human herpesvirus 6 in women. Estimates and 95% confidence bands with the lowest odds as reference points. Multivariable binary logistic models were adjusted for age, years of education, rooted alcohol consumption, physical activity, smoking and drinking status. Note: P-values refer to overall associations.

^d^ Non-linear associations are illustrated in Fig 4.

While the estimates were not significant in men, in women there were noticeable associations between anthropometric measures and varicella-zoster virus; BMI, BAI, WHeiR and WC showed significant odds ratios per one standard deviation in the range of 1.17 to 1.22 with small confidence intervals and E-values for the point estimates between 1.62 and 1.73. The odds ratios for waist circumference (OR = 1.22; CI: 1.06–1.41; P-value = 0.007) as well as waist-to-height ratio (OR = 1.22; CI: 1.05–1.42; P-value = 0.010) were slightly stronger than for BMI (OR = 1.17; CI: 1.02–1.35; P-value = 0.027) and BAI (OR = 1.18; CI: 1.01–1.36; P-value = 0.032), respectively.

Contrary to men, in women three of five anthropometric measures (BMI, BAI and WHeiR) were non-linearly associated with HHV6 seropositivity (Fig 4). Each of them were modelled using restricted cubic splines with 3 knots regarding the AIC. Hereof the U-shaped, but overall non-significant associations (*P*_*BMI*_ = 0.063, *P*_*BAI*_ = 0.069, *P*_*WHeiR*_ = 0.132) revealed increased estimates for participants with either a low or high BMI, BAI or waist-to-height ratio up to $OR_{BMI}^{\left(max\right)}$ = 1.75 (CI: 1.06–2.89), $OR_{BAI}^{\left(max\right)}$ = 1.65 (CI: 1.04–2.61), $OR_{WHeiR}^{\left(max\right)}$ = 1.60 (CI: 0.96–2.67) compared to subjects with BMI = 27.52 kg/m^2^, BAI = 34.96, WHeiR = 0.55, respectively–the reference points with the lowest odds.

### Longitudinal associations

The investigation of associations between the anthropometric measures and seropositivity after a seven-year period showed results comparable to the cross-sectional approach: while again no clear associations were found in men, the same anthropometric measures were significantly associated with the prospective occurrence of VZV seropositivity in women (odds ratios in the range of 1.55 (CI: 1.09–2.19) to 1.78 (CI: 1.26–2.52) per standard deviation) (Table 4). Despite larger confidence intervals due to a smaller cohort including 208 female subjects, all of the appropriate P-values were stronger in the longitudinal approach, concomitant with increasing evidence against unmeasured confounding (E-values between 2.46 and 2.96). A similar effect could be observed in the case of HHV 6, where the P-values in women decreased for all obesity indices (except WHR) below the 10% threshold indicating a trend toward significance.

**Table 4:** Sex-specific associations^a^ of *σ*-standardised anthropometric measures at F4 with the incident or reactivated seropositivity of helicobacter pylori and the human herpesviruses 3, 5 and 6 at FF4 (7-year observation period).

Men	Helicobacter pylori n = 706		Varicella-zoster virus n = 139			Cytomegalovirus n = 590		Human herpesvirus 6 n = 628		
	OR (95% CI)	P-value	OR (95% CI)	P-value	E-value	OR (95% CI)	P-value	OR (95% CI)	P-value	E-value
Body mass index (kg/m^2^)	1.05 (0.74, 1.48)	0.802	1.03 (0.70, 1.53)	0.868		1.22 (0.85, 1.76)	0.278	0.93 (0.78, 1.10)	0.385	
Waist-to-hip ratio	1.23 (0.82, 1.85)	0.311	0.87 (0.57, 1.32)	0.510		non-linear^a^	0.075	0.96 (0.79, 1.17)	0.670	
Waist-to-height ratio	1.18 (0.81, 1.71)	0.386	0.91 (0.60, 1.39)	0.670		1.20 (0.79, 1.83)	0.391	0.92 (0.76, 1.12)	0.411	
Waist circumference (cm)	1.08 (0.76, 1.55)	0.659	0.90 (0.61, 1.34)	0.612		1.14 (0.76, 1.70)	0.524	0.92 (0.77, 1.10)	0.373	
Body adiposity index	1.10 (0.77, 1.57)	0.592	1.01 (0.67, 1.52)	0.978		1.23 (0.83, 1.82)	0.310	0.92 (0.76, 1.11)	0.390	
**Women**	**n = 772**		**n = 208**			**n = 521**		**n = 632**		
Body mass index (kg/m^2^)	1.25 (0.91, 1.72)	0.173	**1.78 (1.26, 2.52)**	**0.001**	2.96	1.05 (0.61, 1.81)	0.868	**1.20 (1.00, 1.44)**	**0.047**	1.69
Waist-to-hip ratio	1.29 (0.90, 1.85)	0.172	1.39 (0.99, 1.95)	0.057		1.72 (0.98, 3.02)	0.057	1.03 (0.86, 1.25)	0.735	
Waist-to-height ratio	1.31 (0.92, 1.86)	0.137	**1.68 (1.18, 2.40)**	**0.004**	2.75	1.20 (0.67, 2.14)	0.545	1.18 (0.98, 1.44)	0.088	
Waist circumference (cm)	1.30 (0.93, 1.82)	0.131	**1.70 (1.21, 2.40)**	**0.002**	2.79	**non-linear**^a^	**0.048**	1.20 (1.00, 1.44)	0.056	
Body adiposity index	1.21 (0.87, 1.70)	0.260	**1.55 (1.09, 2.19)**	**0.015**	2.46	0.72 (0.38, 1.40)	0.336	1.20 (0.99, 1.45)	0.065	

Abbrevations: CI, confidence interval; OR, odds ratio.

^a^ Multivariable binary logistic regression models were adjusted for age, years of education, rooted alcohol consumption, physical activity, smoking and drinking status. E-values of point estimates are presented for significant linear associations (bold).

^b^ Body adiposity index = (hip circumference in cm / height in m^3/2^) - 18.

**Fig 5:**
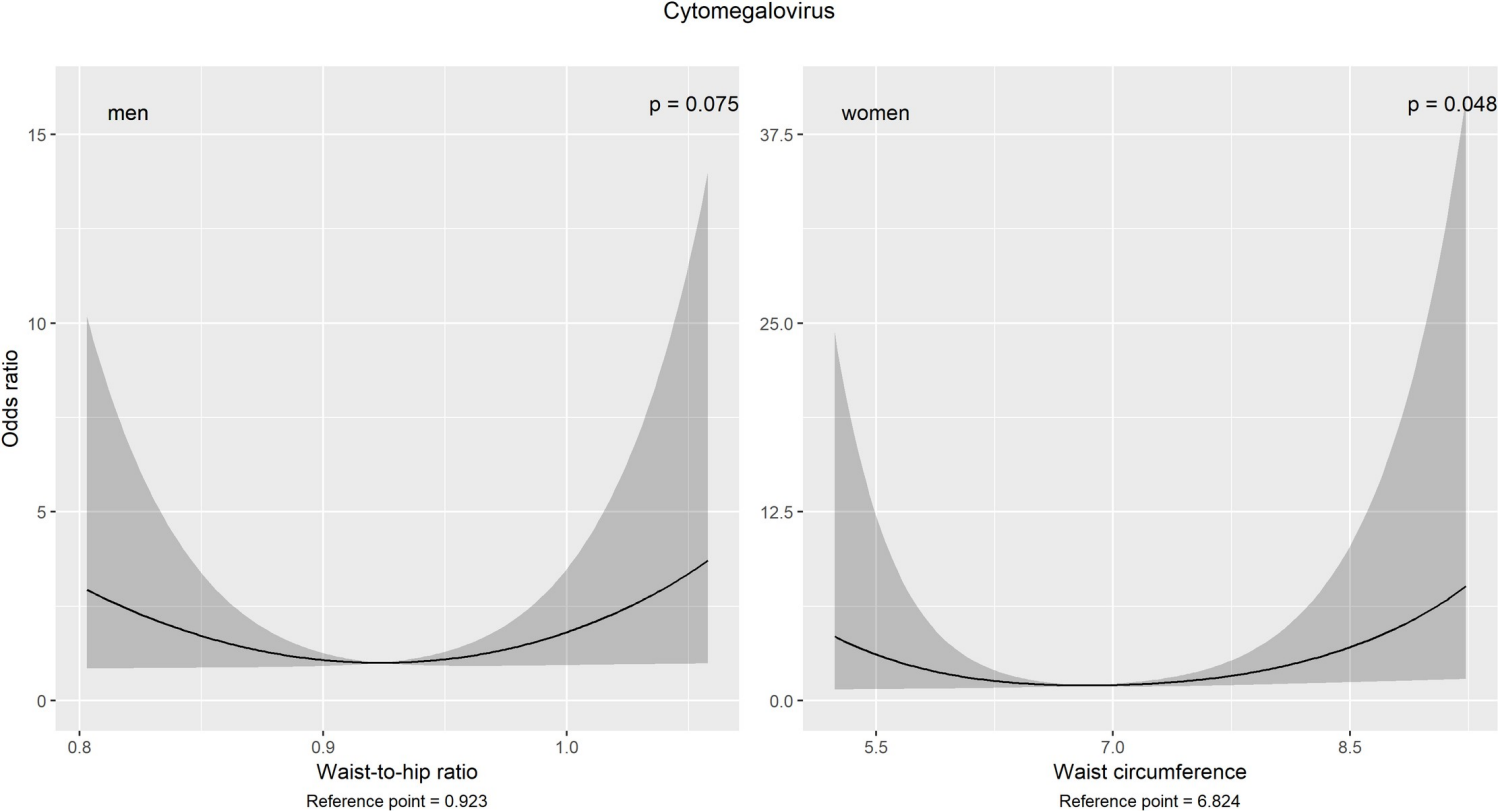
Non-linear associations with the cytomegalovirus-seropositivity after a 7-year period. Estimates and 95% confidence bands with the lowest odds as reference points. Multivariable binary logistic models were adjusted for age, years of education, rooted alcohol consumption, physical activity, smoking and drinking status. Note: P-values refer to overall associations.

^c^ Non-linear associations are illustrated in Fig 5.

Using restricted cubic splines with 3 knots (regarding the AIC as well as sample size and uncertainty of the estimates), U-shaped associations were observed between WHR in men as well as WC in women and the occurrence of CMV-seropositivity (Fig 5). Contrary to the cross-sectional approach, none of the anthropometric measures was modified by age.

Overall, due to only sporadically significant results, no clear evidence for an association between anthropometric measures and longitudinal seropositivity of HP, CMV and HHV 6 in both sexes.

## Discussion

The present population-based study revealed that among women, general and abdominal obesity were associated with both prevalence of seropositivity and the prospective occurrence of seropositivity varicella-zoster virus, since 4 of 5 investigated anthropometric measures (BMI, BAI, WC and WHeiR) showed a significant positive linear relationship. Furthermore, comparing the strengths of associations between BMI, BAI, WC and WHeiR and the varicella-zoster virus seropositivity, no differences were found, indicating that the associations are independent of adipose tissue distribution. Finally, there was a non-significant inverse linear association of the anthropometric measures with the seroprevalence of EBV in both sexes and the trend to linear positive associations with the detection of HHV 6 seropositivity among women. In men, no significant associations between the anthropometric measures and the prevalence as well as occurrence of seropositivity of chronic infections during follow-up were found.

Our results are partly in accordance with findings of other studies [20, 21]. While we could confirm a trend towards a negative association between obesity indices and EBV-seroprevalence and no association with CMV seropositivity, we did not observe a significant relationship of obesity with the seroprevalence of HP. In their cross-sectional study with 985 adult participants, Thjodleifsson et al. [21] reported a significantly positive association between overweight (BMI > 25 kg/m^2^) and IgG antibodies against HP (OR = 1.86; 95% CI: 1.34–2.60). Contrary to this study, we used (based on 3080 subjects) non-linear sex-specific models with inverse exposure-outcome modelling of five obesity measures as exposures and the seroprevalence of infections as outcomes. Our study also differs from that of Thjodleifsson et al. concerning the consideration of alcohol consumption as an additional confounding factor in the models. In addition, other studies on obesity and seroprevalence of HP reported inconsistent results [22, 23]. So far, comparable studies regarding sex-specific associations between obesity and the cumulative exposure, i.e. the presence of antibodies due to past or present infection, to VZV and HHV 6 are missing in the literature. Thus, further investigations are needed to evaluate our findings.

Based on significant exposure-sex interaction terms, our analyses were stratified by sex. This fits also with the well-described differences in total body fat content as well as the specific fat distribution pattern of normal-weight men and women. With increasing body fat content, men are more likely to accumulate adipose tissue in the abdominal region while in women the hip regions may be preferred [24]. Most interestingly, the phenomenon of significant associations in women but not in men was already reported for the human herpesvirus 1 (= human simplex virus 1) [25], and female sex was suggested as an risk factor in this context [26].

BMI is the most common relative body weight measure in this area, which is also used to classify adults into weight categories (underweight, normal-weight, overweight, obese); however, like other body weight indices, the BMI has its strengths and limitations [7]. BMI and BAI describe overall body weight in relation to height (i.e., relative body weight), while WC, WHR and WHeiR consider abdominal fat accumulation. By using all of these anthropometric variables in a standardised way, we were able to, on the one hand, conduct a cross-comparison due to the strength of exposure-outcome associations and therefore could make statements about differences between visceral and overall body fat tissue. On the other hand, these obesity indices could be examined collectively to get a reciprocal verification of each association with increased confidence. Therefore, exposure-outcome associations were considered as significant only if the majority of the anthropometric measures (which reflect different aspects of body fat and its distribution) were significantly associated with the respective outcome. Sporadically observed significant associations were considered spurious due to multiple testing.

Obesity as a multifactorial disease affects a number of physiological processes and pathways that subsequently lead to immune system dysregulation, an impaired immune response and finally to an increased susceptibility to both bacterial and viral infections [1–3]. A metabolic dysregulation of the complex interaction between adipocytes and leukocytes promotes immunodeficiency due to altered secretion of pro- and antiinflammatory factors, including adipokines (e.g. leptin, adiponectin, omentin, resistin and further proinflammatory cytokines), which have an impact on the number of T-cells (altered CD8+/CD4+ ratio), natural killer cell activity as well as antigen presentation by dendritic cells. There are also sexual inequalities within immune surveillance based on sex-specific anatomy, hormonal factors and social behaviour [27]. In addition, there is evidence that sex influences the immune response. On the one hand, women are more resistant to bacterial and viral infections due to overall higher antibody levels as well as greater T cell activation [28], while men seem more susceptible to infectious diseases, attributed to a hormone-dependent expression of cell receptors involved in viral entry [29, 30]. On the other hand, these mechanisms promote an increased susceptibility to chronic inflammatory diseases in women [27]. In our study, sex-specific differences were observed, which need to be clarified in further investigations.

Our prospective cohort study had the advantage of being population-based by including participants of both sexes from a European country. It is one of the largest studies regarding the seropositivity status of chronic infections, which was measured identically via multiplex serology for each of the five infections that were investigated. The prospective design in particular minimizes reverse causality in the sense of the cause-effect chain, which is often an issue in epidemiological studies. The large number of adults with given seropositivity status, in combination with the long-term follow-up of the KORA-study, allowed us to extract a sufficient number of seronegative participants and to investigate the occurrence of primary infection or reactivation of latent infections (except EBV) in a longitudinal approach. However, information on seropositivity certifies the history of infection rather than a current chronic infection. Such a classification contains no information about the time point of infection. Especially VZV and HHV 6 infections happen in childhood, so seropositivity within a seven-year period of adult subjects usually implies the reactivation of latent pathogens when the cellular immune system is compromized [31].

Therefore, our results are not comparable with studies that used measures of a current infection [32, 33]. Furthermore, we had no information on vaccination history, although a vaccination especially for VZV (that only exists since 2004 in Germany) is very unlikely for adult participants in this cohort.

Although we used multiple anthropometric measures to decrease the likelihood of randomly occurring significant results, which can result from multiple testing, all of these measures have the drawback of only being able to approximate true body fat content. A more exact body composition assessment, like Bioelectric Impedance Analysis (BIA), would increase the precision of the results.

To minimise possible bias of the investigated associations induced by residual confounding, we avoided the categorisation of numerical covariates. For age, one of the most influential confounders in our analyses, we used restricted cubic splines with 3 or 4 knots for half of the models instead of categorization, when the conditional relationship with the log odds of an appropriate seropositivity status was non-linear. We estimated that a potential unobserved confounder would need to have a significant OR of at least 1.62 to 1.73 in the cross-sectional analysis or 2.46 to 2.96 in the longitudinal approach to be able to eliminate the significant associations with VZV-seropositivity seen in women. These point estimates, expressed by E-values, are stronger than the associations of all covariates used in the appropriate regression models. Subsequently, an unobserved confounder would have to be stronger than all observed confounding factors, which is highly unlikely. Finally, the missing mechanism within the seropositivity status of chronic infections in our data was independent from other factors, since they could be ascribed to technical issues in the laboratory. Therefore, the MCAR assumption is satisfied and a complete case analysis should not introduce any bias.

## Conclusions

In summary, obesity was neither cross-sectionally nor prospectively significantly associated with seropositivity of HP, EBV, CMV, and HHV 6 in both sexes. However, higher body fat may increase the susceptibility to primary infection or reactivation of a latent VZV-infection among women, independent of body fat distribution. Further studies are needed to elucidate the conditions that predispose individuals to specific infections, or reactivation of infections.

## Abbreviations

AIC: Akaike’s information criterion
BAI: Body Adiposity Index
BMI: Body Mass Index
CI: Confidence interval
HHV: Human herpesvirus
CMV: Cytomegalovirus (HHV 5)
EBV: Epstein-Barr virus (HHV 4)
HP: Helicobacter pylori
IQR: Interquartile range
OR: Odds ratio
SD: Standard deviation
VZV: Varicella-zoster virus (HHV 3)
WC: Waist circumference
WHeiR: Waist-to-height ratio
WHR: Waist-to-hip ratio
